# The possibility of reducing the risk of suicidal attempt in adolescents by practicing Confucian philosophy: a phenomenological study in Vietnam

**DOI:** 10.3389/fpsyg.2024.1449688

**Published:** 2025-01-07

**Authors:** Thien-Vu Giang, Phi-Bao Nguyen-Ngoc, Vinh-Loc Sam, Van-Son Huynh

**Affiliations:** ^1^Psychology Department, Ho Chi Minh City University of Education, Ho Chi Minh City, Vietnam; ^2^Laboratory of Educational Psychology Potential Research Group, Ho Chi Minh City University of Education, Ho Chi Minh City, Vietnam

**Keywords:** Confucian philosophy, Confucianism, self-nurture, neutrality, suicide attempt, family relationship

## Abstract

**Introduction:**

Suicide attempt in adolescents is a concern topic that differs greatly between countries because of its cultural specificity. This article reported on the possibility of reducing the risk of suicide attempts in adolescents by practicing Confucian philosophy.

**Methods:**

In this study, Confucian philosophy is approached as an educational philosophy applied in clinical practice on 12 adolescents who used to attempt suicide. Using a phenomenological study design, we interviewed the selected participants to describe themes surrounding the impact of practicing Confucian philosophy on cognition, emotions, and behaviors related to suicide attempts.

**Results:**

From this framework, we explored the three main findings, which described the journey of reconnecting and repairing family relationships fractured by the negative influence of remnants of Vietnamese Confucianism families. The conservatism and remnants of traditional Confucianism have influenced how Vietnamese parents raise their children, leading to increased risks of suicide as adolescents.

**Discussion:**

The discovered themes greatly contribute to the process of clinical intervention for adolescents who have attempted suicide in countries with similar cultures to Vietnam, or accepted Confucianism as a neutral approach. Above all, the self-nurture and neutral lifestyle were resources that helped the adolescents cope better with their personal problems after participating in this study.

## 1 Introduction

Currently, adolescent suicide is an important and major public health problem (Pelkonen and Marttunen, 2003). Especially since 2021, WHO has pointed out that the COVID-19 pandemic crisis has significantly hindered mental health care services and raised concerns about the increasing number of suicides worldwide, especially among adolescents (Gracia et al., 2021). Suicide attempts and suicides in adolescents involve one of the highest rates of incidence and prevalence in the Western and Eastern (Schilling et al., 2009; Yıldız et al., 2018). There are many risk factors leading to adolescent suicide attempt, however, these factors are different in studies by cultural and social factors, leading to difficult to accurately predict the high-risk groups in multicultural countries (Mars et al., 2019). A better understanding of these factors is essential for improving suicide prevention and intervention strategies. This context requires new clinical intervention strategies in multicultural countries to minimize the risk of increasing suicide attempt, or psychological support for uncompleted suicide, which can contribute to the positive youth development of the adolescents.

From previous studies on suicide or suicide attempts, although no specific solutions were capable of screening or determined a suicidal person, specific risk factors exist (Barbeito et al., 2021; Muehlenkamp and Gutierrez, 2007). Of importance, the lack of most risk factors does not make an adolescent safe from suicide (Vajda and Steinbeck, 2000). Traditional intervention strategies on suicide attempts have been placed in the perspective of risk factors (Hughes and Asarnow, 2013), but the current intervention strategies heavily depend on the practitioner’s expertise and are not unified under a theoretical framework or approach to intervention. Therefore, when researching ways to intervene and prevent suicide attempts, researchers must adopt a personalized approach and clearly state the impact of cultural, social, and professional factors of the researchers, as well as the psychological characteristics and motivations driving adolescent suicide.

In the current study, we focused on exploring adolescent suicide attempts rather than completed suicides due to the risk and recurrence of suicidal behavior in this group. Additionally, during the sensitive period of 1–8 weeks after an adolescent has made a suicide attempt, the likelihood of another attempt is very high and requires support from clinicians, psychologists, or other health care professionals. Accordingly, suicide attempts are included in the broader definition of self-harm, which means self-inflicted physical harm with or without an intention to die, the latter being taken into account (Pelkonen and Marttunen, 2003). The suicide attempt is a quite sensitive topic in many countries and the universal data has not been released widely. This makes it difficult to determine the reputable database of statistics in research projects such as WHO that has been reported, mainly self-reported or local hospital-based reports on this issue (Bromet et al., 2017; Klonsky et al., 2016). Shain et al. (2016) analyzed that when compared to the completed suicide rate, the suicide attempt was 30 times higher. In the United States, the number of suicide attempts in adolescents were reported as high as 100–200 per suicide (Olfson et al., 2017). Previous studies all committed that the increasing numbers of suicide attempts increased the risk of dying (Barbeito et al., 2021; Turecki et al., 2019; Yıldız et al., 2018) and they are the most relevant risk factor for a completed suicide (Jones et al., 2003).

Although in Asia, adolescent suicide is not new, it remains the focus of many prevention and intervention studies because it is a mass trauma that affects the mental health of children and adolescents (Smith et al., 2021). In Asian countries, the common methods of suicide were poisoning, jumping, falling and hanging (Nissirios et al., 2017). Eastern cultural characteristics are more likely to respect the values, attitudes, beliefs and spirituality that have a strong impact on suicide decisions or intentions to continue living of an individual (Paul, 2008). In Christianity, human life is the most valuable and we live to take care of our body and soul. Islam forbid the suicide. Original Buddhism criticizes suicide for any reason because it is considered to go against the Creator. Meanwhile, Buddhist Japan and atheist China were reported having a higher suicide rate, namely up to 17.9 and 25.6% (Bachmann, 2018). Because almost Asian religions opposed to suicide, resulting in a lot of compression and it was transformed into different suicide and suicide attempt consequences. This context led to a lot of different research results of the positive and negative impact of Asian religions to the adolescents’ suicide (Schwalbe, 2022). Therefore, when studying suicide or suicide attempt in this area, researchers must access the religious framework in that country and consider the multi-dimensional impact of this religious ideology to the perception, perspectives and motivation of the adolescents’ suicide.

In Vietnam, suicide and attempted suicide have only recently been studied in the medical sector. Thanh et al. (2005) surveyed 509 patients (with 515 attempted suicide events) and found that the suicide attempters’ mean age was 28.3 ± 12.9 years. Nearly half (48.7%) were aged 15–24. Acute life stressors were the main causes (73.8%) of suicide attempts. Only in some 6% of cases had a psychiatric illness been diagnosed before the suicide attempts. This study suggested that teaching life skills in schools to help children resolve family conflicts was the first step in preventing suicide. Most recently, Luan et al. (2024) conducted a descriptive, cross-sectional study on 307 young people who used drugs aged 16–24 in the community in Hanoi (Vietnamese capital). This study found that the prevalence of suicide risk among young people using drugs was high. Therefore, suicide risk should be screened and monitored in the clinical assessment of this population. In addition, intervention efforts to detect and intervene in adverse events during childhood may be one way to prevent mental health and suicide in later life. In the authors’ study efforts, there are few studies on suicide or attempted suicide conducted in Vietnam due to communication barriers (under the management of the Government) and no human rights ethics committee to approve and certify these studies. Therefore, this area is a research gap in Vietnam.

An important limitation of previous studies is a reliance on cross-sectional studies and the retrospective reporting of both risk factors and suicide-related outcomes. Longitudinal studies adopting an ideation-to-action framework are extremely scarce (Guzmán et al., 2019), and the few existing studies have been done in clinical or atypical samples (Castellví et al., 2017). Within this study, we aim to extend previous works by using a qualitative approach to find out the possibility of reducing the risk of suicide attempts in adolescents by practicing Confucian philosophy – the most popular religion and national educational practice of Vietnamese children and adolescents. Previously, some studies have only examined Confucianism as a risk or preventive factor for mental health problems in adolescents, within the framework of the relationship between Confucianism and Vietnamese parenting styles and educational orientation (Giang et al., 2021; Le and Jin, 2024; Vuong et al., 2018). To the authors’ knowledge, there has been almost no research in Vietnam that considers Confucianism as a clinical practice reference framework for intervening in mental health problems for adolescents and other populations. Therefore, within this framework, our findings will expand the understanding of positive practice of Confucian philosophy in the clinical intervention for adolescents with suicide attempts.

## 2 Conceptual framework

Vietnamese Confucianism has been both a religion and an educational – cultural ideology of Vietnam from the ancient times to the present (Vuong et al., 2018). The current national educational curriculum in Vietnam has subtly selected and integrated Confucian philosophy into educational content aimed at developing students’ competencies and qualities (Oanh, 2021). The core philosophy in Confucian doctrine is lifelong self-learning and self-development.

Confucian philosophy includes two main pillars, the Lower chapter and the Upper chapter, which are both an ideology and a practical guide for believers in self-development, learning, social connection and dedication. The Lower chapter prescribes basic instructions on the theory of ‘self-nurture’, the living philosophy of ‘neutrality’ and the value of social relationships surrounding an individual (family, school, society and country). Meanwhile, the Upper chapter focuses on exploring and explaining the philosophies of the universe and human beings, which guides people to the authentic truth (O'Harrow, 2021). The difference between Vietnamese Confucianism and other religions, even the original Confucianism in China, lies in the methods that the believer practice and maintain religious activities. For Vietnamese Confucians, rituals, worship practices, religious activities or prayers are not necessary. The most important goal that Vietnamese Confucian believers need to accomplish through their ‘life journey’ is to study, read, understand, and reflect on the teachings of Confucius to others. Accordingly, Vietnamese Confucianism exists as the people’s ideology and cultural standard of manner (Vuong et al., 2018). In terms of religious worship, Confucianism does not conflict with the doctrines of other religions. Practitioners of Confucianism in Vietnam, as well as the way Confucianism is practiced in Vietnam, do not require individuals to follow Confucianism exclusively; they can worship Buddhism, Christianity but still practice Confucianism in their work and life. This clearly demonstrates the ‘neutrality’ philosophy of Confucianism, aiming for harmony and balance in perspective, emotion, and practice in social relationships.

Although many studies have discovered and commented on the outdated rules and conservative aspects of Confucianism as a hindrance to human psychological development (Hwang, 2001; Liu, 2021; Rao, 2019), other studies have also proven the positive impact of Confucianism on mental health, even in counseling and psychological therapy practices when practitioners and guides understand the authentic perception of Confucian philosophy (Liu, 2014; Giang and Huynh, 2022; You, 2023). Religious, belief, and spiritual factors have been found to be closely related to adolescent suicide attempts in Asia (Headley, 2023). Confucian philosophy has been found to have a dual influence on the adolescent mental health, including suicide related issues. Confucianism’s emphasis on filial piety and adherence to parental guidance (such as the principle where ‘the father is the principle of the son’) can exert considerable pressure on adolescents (Le and Jin, 2024). The cognitive dissonance between parental expectations and self-affirmation might contribute to familial conflicts, where traditional parental control faces resistance from gradually individualized adolescents’ ways of life due to economic development (Yu et al., 2015). These experiencing severe cognitive dissonance might eventually escalate into the suicidal acts. Also, it is essential to consider that Confucian philosophy might also influence the parental psychological mechanisms at play (Barber, 1996; Bedford and Yeh, 2020; Chuang et al., 2018). Previous studies have indicated that Asian parents often use guilt induction, shame, and love withdrawal as a psychological manipulation tool, which aligns with Confucian values that emphasize familial hierarchy and filial piety (Yu et al., 2015). Therefore, if the adolescent trauma stemmed from this parenting style, practicing Confucian philosophy might have negative effects on their mental health. However, no discoveries have been published regarding this phenomenon in Vietnam – Southeast Asia.

**Figure 1:**
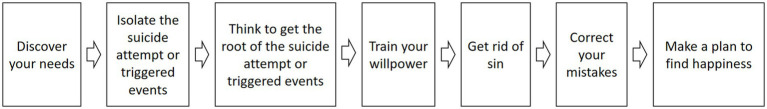
Practicing the self-nurture – cultivation of mentality.

The current study is designed to preliminarily explore the impact of religion, belief, and spirituality on the suicide attempts of Vietnamese adolescents. Therefore, we choose the religious conceptual framework as Confucian philosophy due to its popularity and potential in psychological interventions for adolescents at risk of suicide or attempting suicide. In this setting, we acknowledge this framework limitations and specificity in the context of Asian parenting styles; and using this approach would uncover both positive and negative influences related to suicidal attempts in Vietnamese adolescents. The practical basis of Confucian philosophy is based on the principle of “self-nurture” and “neutrality” (in the Lower part). Accordingly, a suicide attempt is viewed as a meaningful event in the life of the practitioner. To reduce the likelihood of a suicide attempt, the practitioner can sequentially perform 7-steps of self-nurture aiming towards the harmony in their mental health (see Figure 1). These stages were explored in a qualitative study for clinical intervention in a case of sexual-abused Vietnamese Confucian women, with similar cultural contexts, respectively (Giang et al., 2021).

Self-nurture steps align with Slaikeu’s (1989) crisis theory, which emphasized the importance of identifying the triggering event of suicide. This event might stem from traumatic occurrences or developmental transitions (Slaikeu, 1989). The purpose of crisis intervention included integrating the event into the individual’s life, suggesting that there was a solution to the situation. This traumatic triggering event is perceived as a ‘catalyst’ in the individual’s resilience and development process. All individual’s psychological capital or intrinsic development assets are emphasized and promoted to support the process of cognitive change to help individuals adapt more quickly to their trauma. For children and adolescents who have attempted suicide, this crisis intervention protocol is recommended to provide timely and significantly intervention during the ‘window of crisis’ after the suicide attempt (Estrada et al., 2015).

In this study, the authors approached the clinical intervention for adolescents who have attempted suicide based on the theoretical framework of practicing self-nurture (Cultivation of mentality) derived from Vietnamese Confucian philosophy. We aimed to validate and expand our understanding of clinical intervention strategies for suicide attempts in adolescents in the multicultural developing countries. In any multicultural context, psychologists, clinicians or psychiatrists need to be considerate of the client’s religious and cultural framework. The intervention process adhered to ethical guidelines in clinical practice and involved 7-steps of self-nurture with the aim of reducing the risk of suicide attempts among adolescents.

## 3 Methodology

### 3.1 Study design

The current study aimed to explore the possibility of reducing the risk of suicide attempt in adolescents by practicing Vietnamese Confucian philosophy. Because of the life-sensitive essence of our study, we strictly adhered to national medical and research ethics committee and the clinical practice guidelines of the American Psychological Association. The Research Ethics Committee Board in the Social sciences and Humanities of a key university under the Vietnam Ministry of Education and Training approved this study (project code: QD4167-30122022 and QD4111-2712022), under the strict supervision of the Clinical Inpatient Department of the hospitals managing the participants. Moreover, we followed the Declaration of Helsinki when studied about human in this study setting. Permission to access the participants was approved by the hospital directors, clinical therapists, and parents of participants. At this stage of research, it endeavored to explore how these lived experiences (practicing Vietnamese Confucian philosophy) have shaped the current framework and reduced the adolescents’ suicide attempt. The researchers chose hermeneutic methodology in qualitative approach of Heidegger’s perspective for collecting and interpreting the participants’ experience (Nigar, 2020). In this setting, the researchers overcome or suspends prior knowledge to understand deeply the experience of the participants, who were directly attempted suicide. Within this study’s scope, Im et al. (2004) suggested the ‘Cultural integrity’ which was an interpreted framework to gain creditability results when studying the suicide attempt and Confucian philosophy practice experience:

1. ‘Cultural relevance,’ which derived from its key aimed to explore the effect of practicing Confucian philosophy to suicide attempt in adolescents. Within this framework, researchers could identify the possibility of reducing the risk of suicide attempt by practicing Confucian philosophy. The research team’s potential to carry out this study setting was presented in Table 1.
2. ‘Contextuality,’ which based on the researchers’ in-depth and relevant background related to Confucian philosophy and suicide attempt in adolescents. This permitted the researchers to approach participants respectfully. The research team ensured that all members had full understanding and rich experience in researching and practicing Vietnamese Confucian philosophy. No prior relationship between the researchers and the participants were reported and confirmed.
3. ‘Appropriateness,’ which required the translation and interpretation process of participants’ language congruent. In this study, the translators and at least two of the researchers must be influent in both English and Vietnamese, also the Confucianism. The transcripts were proofread by a native researcher who had both knowledge and experience in Confucian philosophy practice and research. The first and second authors made efforts in comparison of translated transcripts with other transcripts to gain conceptual equivalence and credibility. Besides, these authors were responsible for regenerating and analyzing the themes because of their in-depth experience in Confucian practice. To limit subjective analysis, the third and fourth authors took responsible for the critical mass of randomly selected transcripts to triangulate and strengthen the credibility of the findings. After the discussions, the authors refined the themes as these emerged during data analysis until the most credible interpretation of the data was reached.
4. ‘Mutual respect,’ which was gained from the participants’ recruitment. Consent to participate in this study was voluntary. Participants had the right to refuse or withdraw from the study without having to provide any reason, and anytime. They were also allowed to choose the interviews’ time and method (online via Zoom/Google Meet platforms, or on-site at the hospital-clinical office). Participants are fully explained about the content, requirements, risks, rights and responsibilities when participating in the study, confidentially. Next, they were clinically evaluated by an independent (third party) clinical psychologist regarding their mental health status after the suicide attempt. After that, they were received in-depth intervention and supervision by this clinical psychologist throughout the process of participating in interview sessions by researchers. At the same time, the researchers both provided experimental content (clinical explanations and discussions about Confucian philosophical practices) and monitored the clinical intervention process of the independent clinical psychologist. This is established so that the intervention took place in the most effective way. The collected data were asked for permission from the participants to record by taps. As a further measure of respect, interview data after being translated and coded would be sent to the participants to review once to ensure accuracy.
5. ‘Flexibility,’ which was framed through the data collecting period. Because the participants had attempted suicide, the interview space and clinical support had to meet the criteria of privacy, comfort, and a sense of security for them. The participants had the option of refusing to answer or quitting/withdrawing from the study without any explanation. Significantly, none of the participated adolescents refused to interview, or withdrawn from the study.

**Table 1:** The researchers’ backgrounds.

Research team	Occupation	Experience and training
The 1st author	Counselor, clinical psychologist, researcher	More than 5 years of experience practicing counseling, skill training, and conducting the qualitative study. Has 7 years of experience in counseling and intervention for suicide and suicide attempt in adolescents. More than 15 years of religious and spiritual attendance and meditation (in Confucianism, Buddhism, and Christianity).
The 2nd author	Counselor, researcher	More than 2 years of experience in practicing counseling for suicide attempt in adolescents. More than 10 years of practicing Confucian philosophy.
The 3rd author	Clinical psychologist, researcher	More than 10 years of experience in intervention for suicide, suicide attempt and different types of self-harm in children, adolescents and young adults. Currently being a Buddhist and Confucian scholar.
The 4th author	Counselor, researcher	More than 20 years of research and practice experience in school psychology, counseling psychology, Confucianism, Vietnamese ancient and modern culture, human behavior, and social science.

### 3.2 Participants

Based on the study’s scope, participants were Vietnamese adolescents between 12 and 18 years old, who had made the suicide attempt among the last 4–8 weeks. At that time, they must have been receiving the psychotherapy and emergency-supervising at the hospital. Additionally, due to the religious conceptual framework in this study being Vietnamese Confucianism – a religion that values family highly, and since the causes of suicidal attempts among adolescents previously published mostly relate to family issues, we only selected participants who had suicidal attempts due to family conflicts or poor quality of family relationships. Therefore, we contacted clinical psychologists at the hospital who had directly treated the participants to confirm their mental health status before recruitment into this study. Six male and six female adolescents (*M* = 15.2, SD = 2.094) participated in the current study. All of their demographic information was anonymized and denoted by the word ‘A’ combined with the serial number that appeared in the interview data transcripts, e.g., A3 for the third adolescents interviewed (see Table 2).

**Table 2:** The demographic information of the participants.

Participants	Gender	Age	Typical general medical	Specialized psychological care in the current regional health care system	Trigger suicide events
A1	Male	18	Physical and mental injury from overdosing on sleeping pills. Depression disorder.	Psychotherapy and emergency-supervising at the regional hospital. None of clinical psychological intervention.	Family conflict
A2	Male	12	Physical injury from jumping from a building. Bipolar disorder	Psychotherapy and emergency-supervising at the regional hospital. None of clinical psychological intervention.	Family conflict
A3	Male	14	Physical and mental injury from overdosing on sleeping pills. Mood disorder.	Psychotherapy and emergency-supervising at the regional hospital. None of clinical psychological intervention.	Family conflict
A4	Female	16	Internal organ damage from taking pesticides. Mood disorder.	Psychotherapy and emergency-supervising at the regional hospital. None of clinical psychological intervention.	Poor quality of family relationship
A5	Female	17	Internal organ damage from taking pesticides. Depression disorder.	Psychotherapy and emergency-supervising at the regional hospital. None of clinical psychological intervention.	Poor quality of family relationship
A6	Female	17	Physical injury from jumping from a building. Mood disorder.	Psychotherapy and emergency-supervising at the regional hospital. None of clinical psychological intervention.	Family conflict
A7	Female	18	Repeated hand cuts leading to blood loss. Bipolar disorder.	Psychotherapy and emergency-supervising at the regional hospital. None of clinical psychological intervention.	Poor quality of family relationship
A8	Female	12	Physical injury from jumping from a building. PTSD symptoms.	Psychotherapy and emergency-supervising at the regional hospital. None of clinical psychological intervention.	Poor quality of family relationship
A9	Male	14	Physical injury from jumping from a building. PTSD symptoms.	Psychotherapy and emergency-supervising at the regional hospital. None of clinical psychological intervention.	Poor quality of family relationship
A10	Male	14	Repeated hand cuts leading to blood loss. Bipolar disorder	Psychotherapy and emergency-supervising at the regional hospital. None of clinical psychological intervention.	Family conflict
A11	Female	15	Internal organ damage from taking pesticides. PTSD symptoms.	Psychotherapy and emergency-supervising at the regional hospital. None of clinical psychological intervention.	Poor quality of family relationship
A12	Male	16	Repeated hand cuts leading to blood loss. Depression disorder.	Psychotherapy and emergency-supervising at the regional hospital. None of clinical psychological intervention.	Family conflict

In phenomenological study, sample sizes between 6 and 20 individuals are accepted to help the researchers gain a detailed and personalized understanding of the phenomenon (Alase, 2017). Twelve adolescents, who were receiving inpatient psychotherapy at Vietnamese public hospitals, were recruited by purposive sampling. This recruitment was supported by the regional hospitals in Ho Chi Minh City (the largest city in Vietnamese Southern), that had clinical psychology departments. The research team contacted the hospital as both a researcher and a clinical specialist. The process of exchanging information about this study with the hospital representative in accordance with the research ethics and signed a written agreement on cooperation in clinical intervention for adolescents who attempted suicide between the research team and hospital. Participants’ personal information was strictly confidential. The hospital representative was the person in charge of telephone contact with adolescents after discharge from the hospital to ask their opinions about participating in the study, and to obtain permission from their parents. When the adolescents (and their parents) consented to participate, the hospital representative would forward the adolescents’ contact information to the researchers. In addition, participation in the study mean that the participants entered the clinical process. Because the suicide triggering event in these participants was related to the family issues, we provided parents who agreed to have their children participate in the clinical process with a family therapy service, with an independent clinical psychologist participation. This support was recognized as our effort in this study and early intervention process for the participants based on the application of Confucian philosophy to the living environment. Both the adolescents and their parents were provided with adequate information about ethical principles in the clinical practice, including signing a non-suicide commitment with the counselor/clinician (the researcher in charged). After being informed about the research procedure, no participants or their parents refused to participate or withdrew from the study, even though it was their right and they recognized it. This was a positive sign that the participants have the desire to overcome their problems. Throughout the interview process, participants were hospitalized as inpatients and the researchers conducted the interviews face-to-face at the hospital-clinical offices. For participants who chose to be interviewed online, we provided support from hospital social workers in connecting laptops for them to participate in the interview.

### 3.3 Data collection

Twelve in-depth interviews, which lasted for 60 min per interview, were recorded in Vietnamese languages. Semi-structured one-by-one interviews were the researchers’ essential method to collect data in this study. We designed a list of question to explore the participants’ experience during the clinical intervention and how Confucian philosophy influenced and helped them overcome suicide attempt. Interviews took place at a later stage after participants had overcome suicidal attempt during clinical process. They regained confidence in themselves, know how to think positively, manage their emotions and were clinical evaluated as no longer at high-risk of suicide. Every data collection process ensures safety of life and timely intervention from medical staff. Currently, they were asked to interview and recount the change in their perspectives, things that Confucian philosophy has influenced, and how they coped with the triggered event of suicide. We mainly used the open questions to exploit the participants’ experience and created the most comfortable interview atmosphere for them. They were sent a list of question to read and prepare 2 days before the interview date, which included:

1. What do you think when I mention your attempted suicide, recently?
2. Please tell me your experience of practicing the Confucian philosophy during the clinical intervention. How were your feelings?
3. How the Confucian philosophy has helped you reduce the possibility of suicide attempt and overcome your ‘sins’?

Additional questions were designed to deeply probe how the practicing of Confucian philosophy experiences affected the participants’ suicide attempts, such as:

1. Based on your experiences of practicing Confucian philosophy, how will you continue to deal with other mental health problems or the returning of suicidal thoughts, suicide attempt?
2. What do you think about practicing the self-nurture philosophy to strengthen your inner resources toward the goal of confronting past ‘sins’ and self-developing for your future?

In the closing section, participants were asked questions to ensure that they had enough opportunity to share and discuss their experiences:

1. Is there anything else that you would like to talk about that we have not covered?
2. Do you feel that you had a chance to share everything you wanted to?

### 3.4 Data analysis

Given the scope of the present study, thematic analysis was used to analyze and interpret the data deductively. This process was done by familiarizing oneself with the data, generating initial codes, searching for themes, reviewing the themes, and naming the themes (Braun and Clarke, 2006). Cultural integrity was noted and strictly followed by the researchers in this step. The first and second authors transcribed and interpreted the data, then, generated the potential themes. The third and fourth authors were in charge of reviewing these themes to refine and achieve the highest reliability. These steps were strictly followed to ensure the best quality and rigor of thematic analysis (Braun and Clarke, 2006).

As a practicing clinical psychologist, the authors were aware that they must remain neutral during the interviewing. They cannot discuss or response the participants’ experience with their personal perspectives, or even force or impose their perspective on the participants. Although difficult, these authors guarded against providing therapy during the interview. Providing clinical interventions based on Confucian philosophy was the duty of an independent clinical psychologist. The researchers’ task was to provide training and professional supervision to this third-party psychologist.

## 4 Findings

Three major themes were identified in the current study: (1) Create an ‘empty space’ for emotional chaos; (2) Mitigate ‘guilt’ through coping self-efficacy; (3) Recover and social engage by re-establishing religious beliefs.

### 4.1 Create an ‘empty space’ for emotional chaos’

Before receiving psychological intervention, participants reported experiencing emotional chaos following their suicide attempts. Feelings of guilt, regret, sorrow, despair, depression, and sensations of ‘luck’ and ‘gratitude for being alive’ were shared by the participants.

‘I felt guilty for making my parents worried these past days. I was so thoughtless…’ (A2) I could not describe my feelings. It seemed I feel nothing at all after doing that. [jumping off the building]” (A8)

‘My feelings were of gratitude and luck for still being alive. I was foolish to do such a stupid thing.’ (A9)

After receiving intervention through the practice of Confucian philosophy, the adolescents’ internal emotions changed. These changes were mostly described by participants as an ‘empty space’ – a void within their soul that contained and cherished their chaotic emotions. Some adolescents even stated that these empty spaces were how they separated their emotions from painful events or triggers for suicidal thoughts after their suicide attempt. The empty space was a metaphor for how a survivor accepted their failed suicide attempt and gave their associated emotions a ‘rest’ in a safe ‘container.’ These findings were recorded in stages 1 (Discover your needs) and 2 (Isolate the suicide attempt or triggered events) of the 7-step self-nurture process.

‘I realize that I have allowed my guilt to ‘struggle’ within the framework I created. What has passed is already gone, I cannot change the past, but I can change the future if I acknowledge that pain exists.’ (A1)

‘The process of cultivating resources over time had greatly improved my thoughts regarding that action [cutting wrists]. I have reduced hatred towards my body and my family. Something within me has changed… It was an empty space where negative things are stored… It acted like a shield protecting me from sorrow and hatred.’ (A5)

‘I fought that now I could separate different containers when facing unfavorable situations in life. The journey of nurturing internal qualities has made me more tolerant and comfortable with my mistaken actions [suicide attempt]. I no longer blamed myself as much as before…’ (A12)

The self-nurture in the process of practicing Confucian philosophy has strengthened the internal qualities of the participants. This helped participants feel more harmonized in their thoughts when reflecting on the events they have experienced in their lives. It was about loving the body and soul. The frameworks within Confucian philosophy strengthened the cognitive beliefs of each participant regarding self-love and the spirit of personal development in life. Regarding to the solidity of these belief frameworks, the chaotic emotions following the suicide attempts have been gradually controlled by the participants.

### 4.2 Mitigate ‘guilt’ through coping self-efficacy’

This theme was illustrated through clinical intervention results achieved in phases 3 (Think to get the root of the suicide attempt or triggered events), 4 (Train your willpower), and 5 (Get rid of sin). The recovery journey of participants was noted when they began to reflect on the relationship between core beliefs, triggered emotions, and suicidal behavior. During this process, guilt, shame, low self-esteem, etc. – collectively referred to as post-effective trauma – showed signs of diminishing as participants focused on cultivating self-awareness in the here and now.

‘I have noticed a change within myself since I learned to focus more on my thoughts and emotions at the present moment. I knew how to stay calm and distracted my thoughts when suicidal thoughts returned. I could control my actions of self-harm because I realized that no one but myself can do this.’ (A11)

‘I knew I was wrong to choose to give up on life just because of a delay in resolving career orientation conflicts with my father. Fortunately, I got a second chance to continue fulfilling my unfinished plans. I cannot deny my mistakes. I would try to convince my father because, after all, he just wanted me to be happy… This was the first time [after the suicide attempt] I saw my father cry for me… I felt so happy…’ (A4)

Coping self-efficacy was one’s confidence in performing coping behaviors when facing life challenges. During the journey of willpower training and cultivating moral qualities, participants gradually developed coping self-efficacy. Adolescents gained confidence in their ability to self-regulate emotions as a coping strategy when emotions and thoughts related to suicidal ideation resurfaced. Participants experienced a shift in belief, accepting and being ready to face the guilt they had felt [suicide attempt] more than in previous stages. A7 reported:

‘I feel that I have bravely faced the fear when seeing the wounds on my body [injured from jumping]. I can cope with the physical transformations each time I thought about suicidal behavior… I focused on breathing and held on to something solid like a table, chair, bed, wardrobe… I believed that I would be happy when I did not allow myself to give up… I would prove to my parents that they can trust my singing abilities, not the label that I must become a ‘doctor’ according to their thoughts…’ (A7)

The willpower of participants after clinical intervention through practicing Confucian philosophy improved significantly. Participants felt a stronger will to live. They realized the life values that ‘tied’ them to this life. They established a survival goal as a foundation to push back suicidal thoughts and reduced the remorse and guilt for their mistakes. In other words, through self-nurture practice, adolescents learned to express confidence in their coping abilities.

### 4.3 Recover and social engage by re-establishing religious beliefs

After practicing and applying Confucian philosophy to deal with internal conflicts caused by suicide attempts, participants gradually gained more confidence in their ability to cope with adversity. Now, triggering events for suicidal thoughts or feelings of guilt for attempting suicide were viewed with more tolerance and positivity. Adolescents seek to rectify mistakes and adapt to overcome the issues that led to suicidal behavior, aligning with stage 6 (Correct your mistakes) in the self-nurture process. A2 and A10 expressed similar sentiments:

‘I wanted to openly discuss with my mother about my father’s current abusive behavior. I wanted her to leave this house. She stayed because of me, enduring suffering… I cannot selfishly only think about myself… My death was not the solution…’ (A2)

‘I realized I need to train to become more confident. I cannot dwell in past pain. My family was not as happy as I hoped, but I cannot let my future be affected by this. I would learn to care for those I love with unconditional love…’ (A10)

In the later stages of intervention, we observed the resilience in participants through re-establishing missed social relationships and re-structuring the religious faith. Most acknowledged having a broader perspective and being open to different life philosophies and religious beliefs. They gradually reconciled conflicting thoughts and accepted differences from those around them. The philosophy of self-nurture has become their life motto – a new inner resource formed after the clinical intervention. A1, A9, and A12 shared their experiences:

‘Right now, I felt free to be myself. I believed in the teachings of Confucius. I believed that all the bad things would pass once I accepted my flaws. I was grateful for the self-nurture. I have learned a lot. These were the lessons and life philosophies for my future.’ (A1)

‘After my suicide experience, I knew punishment was there for me for trying to kill myself… I felt that I need to do something meaningful for my family as a way to rectify my mistakes.’ (A9)

‘Yes, I felt that I had sinned previously (when I attempted), but through the process of self-reflection to rectify mistakes and learn to develop myself, I felt much happier… I talked more with friends and relatives… In these relationships, I recognized motivation to survive and seek happiness… I believed I would be happy when I forgive myself for my mistakes!’ (A12)

Confucian philosophy, as practiced by the participants, enabled forgiveness for past transgressions [due to their suicide attempt] and efforts to improve their situations, resembling stage 7 (Make a plan to find happiness) in the self-nurture process, where adolescents have confidence in themselves to continue developing towards a brighter future. Religious beliefs, particularly Vietnamese Confucianism, might deter suicide attempts through the social engagement fostered by participants’ efforts. The participants’ self-nurture experiences vividly reflect Confucian ideals: ‘Only oneself can save oneself!’.

## 5 Discussion

This study explored the impact of Confucian philosophy on adolescents’ post-suicide attempt experiences, facilitating their recovery through clinical intervention. All twelve subjects exhibited improved mental health following Confucian-based practice in clinical intervention. Engaging in Confucian philosophical study and practice bolstered the participants’ inner resilience, fostering gradual resilience and a more tolerant perspective toward suicide attempt. Drawing from three main themes extrapolated from participant interviews, a deeper analysis was conducted on the internal transformations of adolescents who attempted suicide within the framework of Confucian philosophy.

Firstly, this study reaffirmed the positive influence of religion on adolescent suicide attempts, which was reported in previous studies (Citlak, 2023; Schilling et al., 2009; Kwak and Ickovics, 2019). Placed within the religious framework explored in the context of Vietnam, a developing Eastern country with a long tradition of religious and spiritual culture, Confucian philosophy has contributed to the process of recovery in the adolescents who attempted suicide. The philosophy of self-nurture and neutral mindset were the two core approaches for applying Confucian philosophy into daily life (Oanh, 2021; Vuong et al., 2018). Since the beginning of intervention by clinical psychologists, participants have been taught and guided to learn according to Vietnamese principles, with positive qualities. Throughout the process of both studying and practicing under the supervision of the psychologists and the research team, participants learnt how to accept and cope with factors that directly influence the act of incomplete suicide. A realistic focus on the senses, behavioral expressions, emotions and thoughts of the participant was encouraged to remain. Unpleasant and mixed positive–negative emotions were named, then they were transformed into an ‘empty space’ visualized in the practitioner’s mind. This process has similarities to mindfulness practice or mindfulness-based interventions for individuals who are in a state of trauma or have recently experienced a suicide attempt (Schmelefske et al., 2022). Subsequently, coping self-efficacy was invoked and reinforced, enabling participants to gradually control suicidal ideation during vulnerable periods (Tran et al., 2023). This pivotal transformation underscored the efficacy of interventions for adolescents attempting suicide during critical periods, expanding insights into their experiences beyond quantitative studies’ limitations. Following overcoming suicidal ideation and emotional barriers, participants reintegrated into previously abandoned social relationships, signifying a stage of recovery and re-establishing religious beliefs. Essentially, social re-connection served as a catalyst for recovery in individuals undergoing trauma and distress (Lin et al., 2022; Reivich et al., 2023). This study advocated for the intrinsic motivation behind social re-connection, namely, the re-establishment of religious beliefs. Religious beliefs, within the context of this study, encompassed Vietnamese Confucianism – a religion blending philosophical principles with others without conflict or exclusivity. The philosophy of moderation guided participants in navigating internal conflicts and biases, progressively enhancing their mental resources and coping with life’s challenges. This resilience process aligned with the seven stages of self-nurture, based on established theoretical frameworks. However, previous findings have indicated that Asian parents often use guilt induction, shame, and love withdrawal to ‘force’ the children obey their command (Yu et al., 2015). The guilt and shame experienced by the participants in this study could also likely be influenced by these cultural and parental practices rooted in Confucian philosophy. Therefore, although the authors acknowledge that the participated adolescents’ coping mechanisms, facilitated by learning Confucian philosophy, aim to transform these feelings into self-efficacy and resilience, as they navigate their internal conflicts and familial expectations, it is also reasonable to include that the guilt and shame at the first hand can be induced by parents. In these cases, a family systems therapy approach is essential to continue the intervention for adolescent.

Secondly, we recognized a positive therapeutic relationship between Confucian philosophy and the underlying causes of adolescent suicide behavior: Family relationships. All participants in this study attempted suicide due to family conflicts and parenting styles. They gradually recovered after suicide attempts through a philosophy focusing on developing psychological resources and improving family relationships. In Confucian philosophy, family is the foundation of education and the spiritual origin of each individual (O'Harrow, 2021). Filial piety is a core value that individuals need to maintain and develop from generation to generation (Rao, 2019). Children are obliged to obey their parents absolutely. Equality of status and decision-making rights of children are always under the authority of parents (Liu, 2014). Despite various studies promoting equality and encouraging positive parenting styles in Vietnam, the authority of parents, filial piety, and the spirit of family education focusing on the values of the ancestral line – the main pillars in the Lower part of Confucian philosophy – still exist subconsciously in Vietnamese families (Tho, 2016). This scenario sets the stage for events leading to suicide attempts, as observed in the participating adolescents. They all responded that they could not live in their families because they were not acknowledged, listened to, respected, supported, and loved by their parents. Some previous studies on adolescent suicide also found similar impacts of family relationship quality on suicidal intentions and attempts (Jones et al., 2003; Paul, 2008; Olfson et al., 2016; Schwalbe, 2022). Other suicide factors such as peer conflict, illness, war, gender discrimination, ethnicity, religion, cultural clashes, etc., previously reported in studies outside Southeast Asia (Barbeito et al., 2021; Klonsky et al., 2016; Turecki et al., 2019; Vajda and Steinbeck, 2000), were not found in this study. Thus, the quality of Vietnamese Confucian family relationships in this study influenced the adolescents’ suicide attempt. However, this conflict was ‘healed’ by the philosophy that drove the participants to despair. By arousing, reinforcing, and interpreting to help participants gain an authentic understanding of the nature of Confucian philosophy, they became aware of the problem of their thinking process. The rule of practicing Confucian philosophy must follow the steps from the lowest level of ‘self-nurture’ to higher levels such as ‘family management,’ ‘state governance,’ and ‘world governance’ (McHale, 2008). Only when individuals understand the nature of this philosophy, they can restructure their thinking and the seeds of recovery begin from there. This finding sharpened the positive impact argument of Confucianism in clinical interventions for cases with family-related trauma based on a correct understanding of Confucian philosophy. However, in some cases where the distress that triggered the adolescent suicide stemmed from the ‘sacrifice’ of filial piety by the parents themselves, they were the ones who were ‘traumatized’, and they wanted their children to be filial as a way of accepting their trauma (Barnes, 2005; Cohen and Mannarino, 2015). At this point, Confucian philosophy would become a barrier that prevents the suicidal vortex/loop at any time in adolescents. Although Confucian philosophy also has certain negative impacts on adolescent suicide motivation, if clinical psychologists pay attention to and focus on the misunderstanding in the client’s mindset process, they will effectively help their clients cope with the long-term effects of suicide attempts.

## 6 Limitations

Although we believe that this study has contributed to the field of the relationship between suicide attempt and religion, we acknowledge some limitations. Firstly, the difference in mindset about religion (except Confucianism) and issues related to the relationship between that religion and suicide attempt has not been addressed. The authors’ predominantly religious perspective is Confucianism, placed in a non-conflicting philosophical relationship with other religions. We have not intensely focused on approaching Confucianism from the standpoint of followers of other religions or those with different religious behaviors in this study. This approach may provoke debates about subjectivity. Secondly, this study primarily focused on the role and potential of Confucianism in recovery and thus did not delve deeply into clinical interventions for the participants. Thirdly, the adolescents participating in this study attempted suicide stemming from the quality of family relationships, while other suicide-activating factors were not addressed. The onset signs related to psychopathology such as depressive disorders, anxiety disorders, PTSD, etc. as well as family systems therapy approaches, or other clinical psychotherapy approaches have not been deeply addressed in this study to focus on clarifying the findings of Confucianism. Therefore, the process of clinical intervention cannot avoid the controversy of applying popular family therapies to support participants. Fourthly, evidence indicated that Confucianism has both positive and negative impacts on adolescents’ suicidal intentions and behaviors. The negative aspects of Confucian philosophy on adolescents’ mental health and family circumstances have not been deeply analyzed. Fifthly, the authors were practitioners and researchers in clinical and counseling psychology; we were not clinicians who provided inpatient treatment for patients who had attempted suicide. Therefore, in conducting this study, we could not avoid the health and medical ethics barriers that previous studies (mainly in the field of medicine) have mentioned. Therefore, without guidance, supervision, and proper adherence to the spirit of Confucianism, individuals undergoing training were at high risk of suicide reactivation. These limitations required attention, especially in clinical practice.

## 7 Conclusion

Through this study, we provided a broader understanding of the possibility of Confucianism in reducing the risk of suicide attempt in adolescents. The three themes describing the process of reducing suicide risk reflected the essence of this phenomenon in the study, which is the journey of reconnecting and repairing family relationships fractured by the negative influence of remnants of Vietnamese Confucianism in family life. The conservatism and remnants of traditional Confucianism have influenced how Vietnamese parents raise their children, leading to increased risks of suicide as adolescents enter adulthood. This mindset needed to be recognized and adjusted to its true nature, as doing so can activate the inner resilience of each adolescent, such as the Confucian principles of self-nurture and neutral living. This was a crucial concern in clinical practice and mental health interventions for adolescents with similar religious or cultural backgrounds. The clinical psychologists could conduct a spiritual or religious belief assessment or guide the clients practice Confucian philosophy authentically. These findings allowed us to adjust the goals of counseling and clinical practice from different psychological theories about the feasibility of combining Confucian philosophy practices. This study has provided a counseling and clinical practice framework for adolescents contemplating suicide based on learning Confucian philosophy. Moreover, this is an area of suicide that the researchers could study, especially in religious/traditional moral cultures (Vietnamese Confucianism) to understand cultural resources available to support the recovery of suicide attempters. We have understood at a deeper level of how Confucian philosophy work in the recovery of suicide attempters in Vietnam. In this regard, the role of Confucianism or Vietnamese Confucianism in the aftermath of a suicide attempt cannot be underestimated.

## Data Availability

The raw data supporting the conclusions of this article will be made available by the authors, without undue reservation.
